# Occurrence and determinants of *Klebsiella* species bloodstream infection in the western interior of British Columbia, Canada

**DOI:** 10.1186/s12879-019-4706-8

**Published:** 2019-12-19

**Authors:** Connor B. Reid, Lisa Steele, Kelsey Pasquill, Elizabeth C. Parfitt, Kevin B. Laupland

**Affiliations:** 1 Department of Medicine, Royal Inland Hospital, Kamloops, British Columbia Canada; 2 Department of Pathology and Laboratory Medicine, Royal Inland Hospital, Kamloops, British Columbia Canada; 3 Department of Intensive Care Services, Royal Brisbane and Women’s Hospital, Brisbane, Queensland Australia; 4 Faculty of Health, Queensland University of Technology, Brisbane, Queensland Australia; 5 Intensive Care Services, Level 3 Ned Hanlon Building, Royal Brisbane and Women’s Hospital, Butterfield Street, Herston, Queensland 4029 Australia

**Keywords:** Bacteremia, Mortality, Incidence, Risk factor, Epidemiology

## Abstract

**Background:**

*Klebsiella* species are among the most common causes of bloodstream infection (BSI). However, few studies have evaluated their epidemiology in non-selected populations. The objective was to define the incidence of, risk factors for, and outcomes from *Klebsiella* species BSI among residents of the western interior of British Columbia, Canada.

**Methods:**

Population-based surveillance was conducted between April 1, 2010 and March 31, 2017.

**Results:**

151 episodes were identified for an incidence of 12.1 per 100,000 population per year; the incidences of *K. pneumoniae* and *K. oxytoca* were 9.1 and 2.9 per 100,000 per year, respectively. Overall 24 (16%) were hospital-onset, 90 (60%) were healthcare-associated, and 37 (25%) were community-associated. The median patient age was 71.4 (interquartile range, 58.8–80.9) years and 88 (58%) cases were males. Episodes were uncommon among patients aged < 40 years old and no cases were observed among those aged < 10 years. A number of co-morbid medical illnesses were identified as significant risks and included (incidence rate ratio; 95% confidence interval) cerebrovascular accident (5.9; 3.3–9.9), renal disease 4.3; 2.5–7.0), cancer (3.8; 2.6–5.5), congestive heart failure (3.5; 1.6–6.6), dementia (2.9; 1.5–5.2), diabetes mellitus (2.6; 1.7–3.9), and chronic obstructive pulmonary disease (2.3; 1.5–3.5). Of the 141 (93%) patients admitted to hospital, the median hospital length stay was 8 days (interquartile range, 4–17). The in-hospital and 30-day all cause case-fatality rates were 24/141 (17%) and 27/151 (18%), respectively.

**Conclusions:**

*Klebsiella* species BSI is associated with a significant burden of illness particularly among those with chronic co-morbid illnesses.

## Background

The *Klebsiella* genus, which including the species *K. pneumoniae,* (including subspecies *pneumoniae* and *ozonae*), *K. oxytoca, and K. variicola* are important human pathogens. *Klebsiella pneumoniae* is second to *Escherichia coli* as the most frequent cause of Gram-negative bloodstream infections (BSI) in both hospital and community settings [1]. *Klebsiella pneumoniae* is an important cause of pneumonia, urinary tract infections, septicemia, and intra-abdominal infections [2, 3]. *Klebsiella pneumoniae* has become increasingly important in recent years, as invasive hypermucoid strains have been linked to pyogenic liver abscesses most notably in Taiwan and Korea [4, 5], and there has been an upswing in extended spectrum-β-lactamase (ESBL) producing strains worldwide [6–8]. Species other than *K. pneumoniae* are overall less frequent and have been associated with conditions including BSIs, urinary tract infections, soft tissue infections, and respiratory tract infections [9–11].

Population-based studies minimize a number of important biases and have been recognized as optimal designs to evaluate the epidemiology of BSIs [12]. However, to our knowledge only three previous studies have evaluated *Klebsiella* species BSI in non-selected populations [1, 13, 14]. Furthermore, only one of these has quantified the risks for development of *Klebsiella* species BSI related to specific co-morbid illnesses [1]. The objective of this study was to determine the incidence and risk factors for acquiring *Klebsiella* species BSI among residents of a non-selected Canadian population.

## Methods

### Study population & surveillance

Population surveillance was conducted in the western interior region of British Columbia, Canada (2016 population 182,422) as previously described [15]. The regional microbiology laboratory at the Royal Inland Hospital in Kamloops identified all residents in the selected area with *Klebsiella* species BSI. A senior infectious disease consultant (KBL) then performed a case-by-case chart review to abstract clinical information. The Charlson Comorbidity Index (CCI) was used to classify comorbid illnesses [16]. This study was granted a waiver of individual informed consent by the Interior Health Research Ethics Board (file 201,314,052-I).

### Laboratory procedures and definitions

The BacT/Alert 3D System (bioMerieux, France) was used for blood culturing. Draw of two sets of blood cultures consisting of aerobic/anaerobic bottle pairs from different sites was standard. Organisms were isolated and speciated by examining lactose-fermenting mucoid colonies on MacConkey agar plates. Oxidase tests and MALDI-TOF were then used, along with Gram stain results, for species identification. For the purposes of this study, *K. pneumoniae* included those identified as *K. pneumoniae* and *K. pneumoniae subspecies pneumoniae* or *ozonae*.

Incident *Klebsiella* species BSI was established by the first growth from one or more blood culture sets. Repeat positive cultures within 30 days were deemed be of the same episode. Those reoccurring within 30–365 days were classified as incident if the index episode resolved following treatment. Contaminants were excluded and clinical significance and determinants were established by a review of all information in the electronic health record. Hospital-onset BSI were those where the incident blood culture was drawn 48 h or more and community-onset BSI less than 48 h after acute care hospital admission [17]. Community-onset BSIs were sub-classified as either community- or healthcare-associated using the Freidman et al definitions [18].

### Statistical analysis

Stata version 15 (Stata Corp, College Station, TX) was used for all analyses. Fisher’s exact test was used to examine differences in proportions among categorical data. Skewed continuously distributed variables were described using medians with inter-quartile range (IQR) and were compared using the Wilcoxon-Mann-Whitney test. Incidence rates were expressed as annual rates per 100,000 resident population and calculated using census data [19]. Risks were reported as incidence rate ratios (IRR) with 95% confidence intervals (CI) and were calculated using as numerator and denominator the incidence with and without a factor, respectively. Selected co-morbid illnesses were assessed as risk factors for the development of *Klebsiella* species BSI. Denominator data was obtained from local sources [20, 21], provincial and national surveys [22–28], and studies published elsewhere [29]. *P*-values less than 0.05 were deemed to represent statistical significance.

## Results

### Incidence

During the seven years of surveillance, 153 incident BSI isolates of *Klebsiella* species were identified. In two cases infections had two different incident *Klebsiella* species concomitantly isolated and these included one patient with *K. oxytoca* and *K. pneumoniae* while another with *K. oxytoca* and *K. variicola* co-infection. There were therefore 151 incident *Klebsiella* species BSI episodes among 139 regional residents for an overall annual incidence rate of 12.1 per 100,000 population per year. The incidence of *K. pneumoniae* and *K. oxytoca* were 9.1 and 2.9 per 100,000 population per year, respectively. The remaining two incident isolates were *K. variicola.* Eleven patients had a second and one patient had a third episode of *Klebsiella* species BSI during the surveillance period. Twenty-four cases (16%) were hospital-onset, 90 (60%) were healthcare associated, and 37 (25%) were community-associated.

**Fig. 1:**
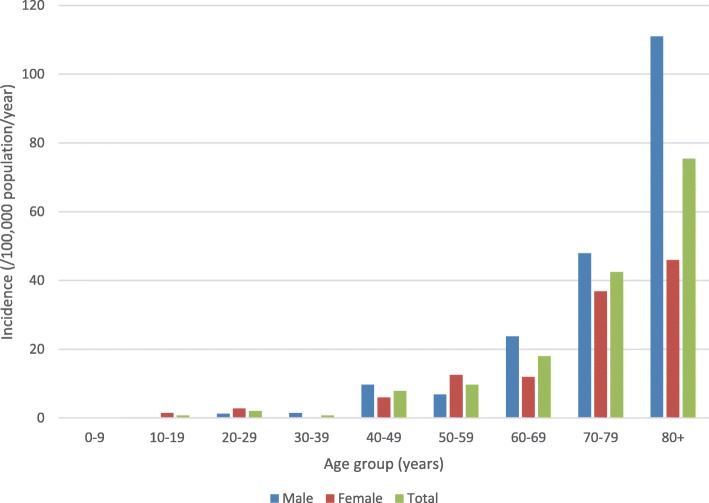
Age and gender specific incidence of *Klebsiella* species bloodstream infection

The annual incidence varied during the study years as shown in Fig. 1. The first five years of the study demonstrated moderate year-to-year variability ranging from 6.8 to 11.7 per 100,000 annually but there was then a marked increase during the fifth and six years of the study (Fig. 1). Although both species increased in incidence in the latter years of the study this was predominantly related to *K. pneumoniae* BSI in the last study year.

### Risk factors and predisposing conditions

**Fig. 2:**
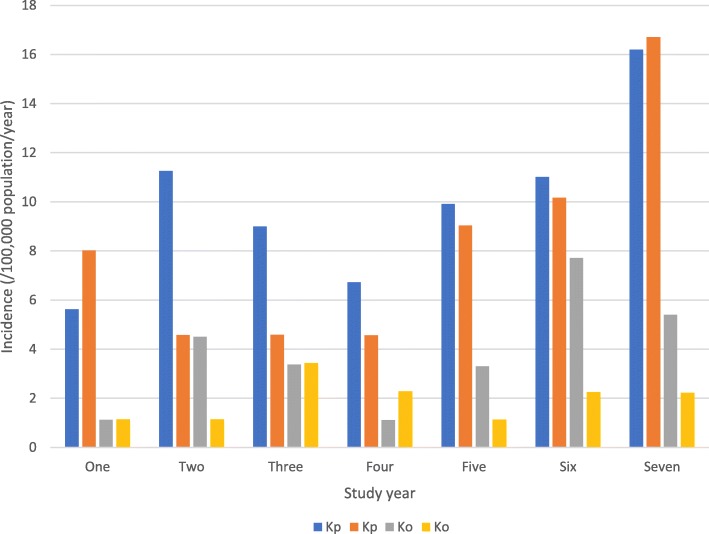
Incidence of *Klebsiella* species bloodstream infection over time (Kp = *Klebsiella pneumoniae*; Ko = *Klebsiella oxytoca*)

The median patient age was 71.4 (IQR, 58.8–80.9) years and 88 (58%) were males. No cases were observed among those aged less than 10 years and the incidence increased with older age as shown in Fig. 2. Males were at higher risk but this was not statistically significant (14.0 vs. 10.1 per 100,000; IRR, 1.4; 95% CI, 1.0–1.9; *p* = 0.06).

The median CCI was 2 (IQR, 1–4). Twenty-eight (19%) patients had a CCI of zero, and sixty-two (41%) had scores of 1–2, thirty-two (21%) 3–4, and twenty-nine (19%) had five or more. A number of co-morbidities were examined as risks for development of a *Klebsiella* species BSI within the population and these are shown in Table 1.

**Table 1:** Co-morbid medical factors associated with risk for development of *Klebsiella* species bloodstream infection

Factor	Estimated prevalence in western interior	Cases	Incidence with factor (per 100,000 population per year)	Incidence without factor (per 100,000 population per year)	IRR (95% CI)	*P*-value
CVA (age 20+)	2.6%	16	81.1	13.9	5.9 (3.3–9.9)	< 0.0001
Renal (age 20+)	3.1%	18	58.8	13.8	4.3 (2.5–7.0)	< 0.0001
Cancer*	4%	45	36.1	9.4	3.8 (2.6–5.5)	< 0.0001
CHF (age 20+)	2%	10	50.1	14.5	3.50 (1.6–6.6)	0.0012
Dementia (age 40+)	3%	12	57.5	19.9	2.9 (1.5–5.2)	0.002
Diabetes	9%	31	27.6	10.6	2.6 (1.7–3.9)	< 0.0001
COPD (age 35+)	10%	30	39.2	16.8	2.3 (1.5–3.5)	0.0002
MI (age 20+)	8%	13	16.5	15.1	1.1 (0.6–1.9)	0.7
PVD (age 40+)	4%	3	10.1	20.1	0.5 (0.1–1.5)	0.2
Liver	10.0%	15	12.0	12.1	1.0 (0.5–1.7)	1.0
Peptic Ulcer	4.2%	10	24.1	14.8	1.6 (0.8–3.1)	0.2
Rheumatic Disease (age 20+)	2.9%	7	24.5	14.9	1.6 (0.6–3.5)	0.2

* Cancer includes solid tumors, leukemia, and lymphoma. Incidence rate ratio (IRR); cerebrovascular accident (CVA); congestive heart failure (CHF); chronic obstructive pulmonary disease (COPD); myocardial infarction (MI); peripheral vascular disease (PVD)

### Clinical foci and microbiology

The most common focus of infection was intra-abdominal or genitourinary as shown in Table 2. Although *K. pneumoniae* was much more likely to be of hospital-onset as compared with *K. oxytoca* BSI (23/113; 20% vs. 1/35; 3%; relative risk 7.1; 95% confidence interval 1.0–50.9; *p* = 0.016), otherwise the demographics and clinical features were similar (Table 2). Although there was a high rate of resistance to ampicillin, most isolates overall were susceptible to ceftriaxone, ciprofloxacin, gentamicin, and co-trimoxazole (Table 2). As compared to *K. pneumoniae*, *K. oxytoca* had a significantly reduced rate of susceptibility to cefazolin (Table 2).

**Table 2:** Clinical features of *Klebsiella* species bloodstream infection

Factor	*K. pneumoniae* (*n* = 113)	*K. oxytoca* (*n* = 35)	p-value
Median age (IQR)	72.4 (62.4–81.3)	64.3 (52.4–76.1)	0.07
Male	62 (55%)	24 (69%)	0.2
Acquisition			0.012
HO	23 (20%)	1 (3%)	
HA	61 (54%)	27 (77%)	
CA	29 (26%)	7 (20%)	
Focus			0.68
Primary/no focus	12 (11%)	4 (11%)	
Bone and joint	2 (2%)	1 (3%)	
Soft tissue	3 (3%)	1 (3%)	
Respiratory	14 (12%)	1 (3%)	
Cardiovascular	2 (2%)	1 (3%)	
Intra-Abdominal	46 (4%)	15 (43%)	
Genitourinary	35 (31%)	12 (34%)	
Antibiotic susceptible			
Ampicillin	2/111 (3%)	1/35 (3%)	0.6
Cefazolin	95/102 (93%)	9/35 (26%)	< 0.0001
Ceftriaxone	72/72 (100%)	29/30 (97%)	0.1
Ciprofloxacin	107/108 (99%)	34/35 (97%)	0.4
Gentamicin	111/111 (100%)	35/35 (100%)	
Co-trimoxazole	100/104 (96%)	32/34 (94%)	0.6
Median CCI (IQR)	2 (1–4)	1.5 (1–3)	0.46

* one case of concomitant *K. pneumoniae* and *K. oxytoca* bloodstream infection excluded from comparative analysis. Interquartile range (IQR); hospital onset (HO); healthcare-associated (HA); community-associated (CA); Charlson Comorbidity Index (CCI)

### Hospital admission and outcome

One hundred and forty-one (93%) patients were admitted to hospital for a median hospital length stay of 8 (IQR, 4–17) days. The in-hospital and 30-day all cause case-fatality rates were 24/141 (17%) and 27/151 (18%), respectively. The 30-day all cause case-fatality rates were 20% (23/113) and 9% (3/35; *p* = 0.1) for *K. pneumoniae* and *K. oxytoca*, respectively.

## Discussion

In this study, we observed that *Klebsiella* specie*s* are frequent causes of BSI. The incidence rate of *Klebsiella* species BSI was 12.0/100,000, and a number of co-morbid medical conditions were associated with significantly increased risk. Furthermore, one in five patients suffering from *Klebsiella* species BSI died within 30 days of diagnosis. *Klebsiella* species BSI cause a significant burden of illness in our population.

To our knowledge, there are only three previous population-based studies published for comparison [1, 13, 14]. Meatherall et al reported on 640 episodes in the Calgary area of Canada during the years 2000 to 2007 and found an incidence rate of for *K. pneumoniae* of 7.1/100,000 [1]. Pepin reported an incidence of 18.7/100,000 in Sherbrooke, Canada, between the years 1997 and 2007, with 411 episodes identified in that time [14]. Finally, Al-Hasan reported 127 episodes in Olmsted County, Minnesota, between the years 1998 and 2007, with an annual incidence rate of 11.7/100,000 for *Klebsiella* species BSI, and 9.7/100,000 for *K. pneumoniae* specifically [13]. The cumulative data from North American population-based studies to date indicate that the incidence rate for *Klebsiella* species BSI is comparable to our observed rate of 12 per 100,000. Individual differences between studies may potentially be explained in part by differences in demographics, rates of culturing, and distribution of risk factors in these populations.

A number of studies have identified co-morbidities most notably cancer and diabetes as risk factors for development of *Klebsiella* species BSI [1, 14, 30–34]. However, with the exception of the population-based investigation reported by Meatherall et al, these studies have been within selected cohorts that are at significant potential risk for bias. In their study from Calgary, Meatherall et al found a number of determinants and that solid organ transplantation, chronic liver disease, dialysis, and cancer were the most important risk factors for development of *K. pneumoniae* BSI [1]. We also observed that renal disease and cancer were significant risk factors for *Klebsiella* species BSI but notably we did not find that liver disease significantly increased the risk. While diabetes was found to be a significant risk factor in both of our studies, after considering the high prevalence among controls in the population the magnitude of this risk was relatively low compared to other co-morbidities (Table 2). There are many reasons why observed risk factors may be different in populations and may be related to the specific populations under evaluation and the differences in study methodologies and definitions.

While this study benefits from the population-based design there are some limitations that merit discussion. First, it is possible that we could have failed to ascertain cases that occurred among residents of our region seeking health care outside our region. Given that almost all tertiary services are available in our area, we suspect that this represents a small number but do not have actual empiric data to justify this claim. Second, a diagnosis of a BSI requires a positive blood culture and as such is related to the decision of clinicians to draw a specimen for testing. This is a limitation inherent to all BSI studies. Third, in establishing comorbid illnesses we did not have individual patient level data on co-morbid illnesses on residents of the region that did not develop BSI and therefore had to estimate prevalence rates in our calculations. A degree of imprecision therefore exists in our determinations and the magnitude of the risk factors reported should be interpreted accordingly. Finally, because of the lack of individual co-morbidity data on controls we were not able to determine independent risk factors for acquisition using multivariable statistics.

## Conclusion

In summary, our study documents the major burden of *Klebsiella* species BSI in our region and is a significant addition to the small number of previous population-based studies conducted in other areas in Canada and the United States. We further identify and confirm that a number of co-morbidities are risk factors for these infections. Further population-based studies conducted in other regions outside of North America are warranted.

## Data Availability

Ethics board approval agreement precludes public sharing of data from this study. Datasets may be made available on reasonable request by contacting the corresponding author.

## Abbreviations

BSI: Bloodstream Infection
CCI: Charlson Comorbidity Index
CHF: Congestive heart failure
CI: Confidence Interval
COPD: Chronic Obstructive Pulmonary Disease
CVA: Cerebrovascular accident
ESBL: Extended Spectrum-Beta-Lactamase
IQR: Inter-Quartile Range
IRR: Incidence-Rate Ratio
MALDI-TOF: Matrix Assisted Laser Desorption/Ionization – Time Of Flight
MI: Myocardial Infarction
PVD: Peripheral vascular disease
RIH: Royal Inland Hospital
